# Sustainable Rubber Solutions: A Study on Bio-Based Oil and Resin Blends

**DOI:** 10.3390/polym17152111

**Published:** 2025-07-31

**Authors:** Frances van Elburg, Fabian Grunert, Claudia Aurisicchio, Micol di Consiglio, Auke Talma, Pilar Bernal-Ortega, Anke Blume

**Affiliations:** 1 Elastomer Technology & Engineering, Department of Mechanics of Solids, Surfaces & Systems (MS3), Faculty of Engineering Technology, University of Twente, Drienerlolaan 5, 7522 NB Enschede, The Netherlands; f.grunert@utwente.nl (F.G.); a.g.talma-1@utwente.nl (A.T.); m.d.p.bernalortega@utwente.nl (P.B.-O.); 2 Bridgestone EU NV/SA, Italian Branch–Technical Center, Via del Fosso del Salceto 13/15, 00128 Rome, Italy

**Keywords:** plasticizers, oils, resins, tire tread, sustainability

## Abstract

One of the most important challenges the tire industry faces is becoming carbon-neutral and using 100% sustainable materials by 2050. Utilizing materials from renewable sources and recycled substances is a key aspect of achieving this goal. Petroleum-based oils, such as Treated Distillate Aromatic Extract (TDAE), are frequently used in rubber compounds, and a promising strategy to enhance sustainability is to use bio-based plasticizer alternatives. However, research has shown that the replacement of TDAE oil with bio-based oils or resins can significantly alter the glass transition temperature (T_g_) of the final compound, influencing the tire properties. In this study, the theory was proposed that using a plasticizer blend, comprising oil and resin, in a rubber compound would result in similar T_g_ values as the reference compound containing TDAE. To test this, the cycloaliphatic di-ester oil Hexamoll DINCH, which can be made out of bio-based feedstock by the BioMass Balance approach, was selected and blended with the cycloaliphatic hydrocarbon resin Escorez 5300. Various oil-to-resin ratios were investigated, and a linear increase in the T_g_ of the vulcanizate was obtained when increasing the resin content and decreasing the oil content. Additionally, a 50/50 blend, consisting of 18.75 phr Hexamoll DINCH and 18.75 phr Escorez 5300, resulted in the same T_g_ of −19 °C as a compound containing 37.5 phr TDAE. Furthermore, this blend resulted in similar curing characteristics and cured Payne effect as the reference with TDAE. Moreover, a similar rolling resistance indicator (tan δ at 60 °C = 0.115), a slight deterioration in wear resistance (ARI = 83%), but an improvement in the stress–strain behavior (M300 = 9.18 ± 0.20 MPa and Ts = 16.3 ± 0.6 MPa) and wet grip indicator (tan δ at 0 °C = 0.427) were observed. The results in this work show the potential of finding a balance between optimal performance and sustainability by using plasticizer blends.

## 1. Introduction

Tire manufacturers aim to reduce their carbon footprint and even become carbon-neutral by 2050 [1]. One of the approaches to achieve this is the transition to using 100% sustainably produced raw materials. These sustainable materials are obtained from renewable sources or recycled substances, which reduces the CO_2_ footprint compared to fossil-based materials [2,3]. Due to the increasing interest in using sustainable materials in tire applications, the availability of scientific papers about the influence of these ingredients in rubber materials is also progressively increasing. Various review articles have been published in the last decade, which together provide an overview of the many sustainable polymers, fillers, plasticizers, and other additives investigated in rubber compounds [4,5,6,7,8,9,10].

One of the ingredient groups under investigation is the plasticizer. Together with the rubber polymer and reinforcing filler, plasticizers take up a large part of the rubber compound. In conventional rubber formulations, petroleum-based plasticizers are added to improve processing and increase the flexibility of the rubber compound [11]. The petroleum-based oil Treated Distillate Aromatic Extract (TDAE) is commonly used in tire tread compounds [12]. A previous study [13] showed that replacing TDAE with various vegetable oils shows potential, but certain material properties exhibit degradation. An important factor contributing to this deterioration in properties is the decrease of the glass transition temperature (T_g_) to lower temperatures. This decrease in T_g_ was widely described in the literature when plant-based oils were used in rubber compounds [8].

Another material that has gained increasing interest in the tire industry is resin [14]. A resin is a low molecular weight (800–4000 g/mol) polymeric material, either in liquid or solid state at room temperature, depending on the softening point [15]. Various resins are available, with differences in chemical structure, molecular weight, and softening point. Resins can be derived from fossil feedstock (e.g., coumarone-indene and hydrocarbon resins) or natural sources (e.g., rosin and polyterpene resins) [15]. These materials act not only as tackifiers but also as softeners and plasticizers. Bernal-Ortega et al. investigated the use of hydrocarbon resins [16] and bio-based resins [17] as replacements for TDAE in simplified tire tread compounds. Based on these studies, it can be concluded that resins with a high softening point and glass transition temperature of the pure resin elevate the T_g_ of the rubber compound, in contrast to most vegetable oils that shift the T_g_ to lower temperatures. This phenomenon occurs when the resin and polymer used are compatible [15].

To achieve optimum performance in the rubber compound, a sustainable alternative to the commonly used petroleum-based plasticizer that results in a similar glass transition temperature is needed. A possible solution for this might be using oil and resin plasticizer blends. The practice of blending oils with resins has been addressed in the literature. Lopitaux [18] described a rubber compound containing 1,2-cyclohexane dicarboxylate diester and a resin. Although similar in-rubber properties were observed, the glass transition temperatures of the vulcanized materials were not analyzed. In another patent, the combination of vegetable oil with resin in rubber is claimed [19]. The experimental example of this patent shows silica-filled SBR/BR compounds with various combinations of petroleum-based oil, soybean oil, and traction resin. Compared to the compound with only petroleum-based oils, the plasticizer blends result in improved wet traction, a similar or worse abrasion resistance, and a slight deterioration in rolling resistance, but a clear improvement in tear strength. However, the T_g_ values of the various compounds were not reported as well.

In this study, the use of plasticizer blends comprising a di-ester oil and a cycloaliphatic hydrocarbon resin as alternatives to TDAE is proposed. It is hypothesized that blending the bio-based oil with resin will shift the glass transition temperature to values more comparable to compounds with TDAE, thereby positively influencing the final material properties of the vulcanized compound. In the first section, the cycloaliphatic di-ester oil Hexamoll DINCH is blended with the Escorez 5300 resin in various ratios to identify the optimal oil-to-resin blend ratio. A detailed evaluation of various plasticizer blend ratios is expected to reveal new trends and correlations in rubber performance properties. The second section explores the use of different di-ester oils (Hexamoll DINCH, Palatinol 10P, and Plastomoll DOA) combined with Escorez 5300 in 50/50 blends, to assess the effects of various chemical structures present in the oils. High concentrations (80 phr) of the plasticizer blends are evaluated to gain a deeper understanding of the effects of replacing TDAE with plasticizer blends on various properties. Because the three selected di-ester oils can be created with the BioMass Balance approach, making these oils up to 100% bio-based [20,21,22] and reducing the CO_2_ footprint by approx. 60% [23]. Therefore, replacement of the petroleum-based TDAE with one of these di-ester oils will improve the sustainability of the final rubber compound.

## 2. Materials and Methods

### 2.1. Materials

The polymers used in this study were Styrene Butadiene Rubber (S-SBR) (SPRINTAN^®^ SLR 4601, styrene content 21%, molecular weight = 450 kg/mol) (Synthos Schkopau GMBH, Schkopau, Germany) and Butadiene Rubber (BR) (Buna^®^ CB24, cis-1,4 content > 96%, molecular weight = 620 kg/mol) (Arlanxeo, Dormagen, Germany). ULTRASIL^®^ 7000 GR (specific surface area (CTAB) 160 m2/g) and bis(triethoxysilylpropyl) disulfide (TESPD) were supplied by Evonik Industries (Wesseling, Germany), and used as highly reinforcing filler and coupling agent, respectively. The curing package consisted of the curing activators zinc oxide (ZnO) (Umicore Zinc Chemicals, Angleur, Belgium) and stearic acid (Emery Oleochemicals GmbH, Düsseldorf, Germany), sulfur (Zolfindustria, Trecate, Italy), the primary accelerator N-tert-butyl-benzothiazole sulfonamide (TBBS) (General Química S.A., Lantarón, Spain) and the secondary accelerator N,N′-diphenylguanidine (DPG) (MLPC International (Arkema Group), Rion-des-Landes, France).

Treated Distillate Aromatic Extract (TDAE) (Hansen & Rosenthal, Hamburg, Germany) was used as a plasticizer in the reference compound. Additionally, the di-ester oils Hexamoll^®^ DINCH, Palatinol^®^ 10P, and Plastomoll^®^ DOA (BASF, Ludwigshafen am Rhein, Germany) were evaluated, as well as the resin Escorez™ 5300 (ExxonMobil, Houston, TX, USA). The di-ester oils were selected based on their availability, potential of being fully sustainable, and saturated nature. Because these oils contain no double bonds in their tail structures, less interference with other rubber ingredients is expected [13]. The Escorez 5300 resin was evaluated in a previous study [16] and showed promising results on the tire performance. Moreover, this resin is widely available and is expected to be compatible with the selected di-ester oils and the used polymers, due to the similarities in Hansen Solubility Parameter (HSP). The HSP values, as well as the molecular weight (Mw), density (ρ), glass transition temperature (T_g_) and chemical structure of the plasticizers are summarized in Table 1. The rubber formulations of the various rubber compounds are shown in the introductions of the separate chapters.

### 2.2. Mixing Procedure

The Brabender Plasticorder 350S (Duisburg, Germany), an internal mixer with a chamber volume of 390 cm^3^, was used to mix the rubber compounds, according to the mixing procedure displayed in Table 2. After each mixing stage, the compounds were sheeted out on a Polymix 80 T two-roll mill (Schwabenthan, Berlin, Germany).

### 2.3. Analytical Testing

#### 2.3.1. Vulcanization

To determine the optimal vulcanization time, the Rubber Process Analyzer TA Elite (TA Instruments, New Castle, DE, USA) was employed. A frequency of 1.667 Hz and a deformation of 6.98% were applied, with the temperature maintained at 160 °C. The curing behavior was monitored for one hour. The time to achieve 90% conversion (t90) was subsequently utilized to cure 2 mm thick test samples for stress–strain and dynamic mechanical analysis measurements. For the 6 mm thick abrasion, and the 12 mm thick hardness specimens, the samples were cured for an additional 3 and 5 min respectively, to ensure that the compounds were cured entirely. The rubber samples were vulcanized at 160 °C under a pressure of 100 bar using a Wickert WLP 1600 hydraulic press (Wickert, Landau in der Pfalz, Germany).

#### 2.3.2. Cured Payne Effect

The Rubber Process Analyzer TA Elite (TA Instruments, New Castle, DE, USA) was utilized to determine the cured Payne effect. Samples were initially cured at 160 °C within the device, according to their t90 values. Immediately following this curing process, the storage modulus (G′) was measured at 100 °C. Strain sweeps ranging from 0.1% to 100% were applied to the cured samples at a frequency of 1.667 Hz. The difference between 0.56% and 100% strain was calculated and used as the cured Payne effect.

#### 2.3.3. Hardness

The hardness of the compounds was evaluated using a Zwick 3150 hardness tester (Zwick, Ulm, Germany) according to ISO 48-4 [25]. Measurements were performed on the Shore A scale.

#### 2.3.4. Stress–Strain Behavior

The stress–strain behavior of the compounds was measured using the Universal Mechanical Tester Zwick Z01 (Zwick, Ulm, Germany) in accordance with ISO 37 [26] (die type 2). A crosshead speed of 500 mm/min was employed, and the tensile tests were conducted at room temperature. Five samples of each compound were tested, and the average values of these measurements were analyzed. Additionally, the median values for each compound are presented in stress–strain graphs.

#### 2.3.5. Dynamical Properties

Dynamic mechanical analysis was conducted using the Gabo-Netzsch Eplexor tester (Netzsch, Germany). Rectangular samples with a thickness of 2 mm and a width of 5.5 mm were measured in tension mode. The analysis utilized a static strain of 0.3% and a dynamic strain of 0.1%. The test was performed over a temperature range from −80 to 80 °C with a heating rate of 2 °C/min and a dynamic load frequency of 10 Hz.

#### 2.3.6. DIN Abrasion

The DIN abrader Montech ABR 3000 Rubber Abrasion Tester (MonTech USA GmbH, Columbia, IN, USA) was selected to analyze the abrasion resistance of the vulcanized in accordance with ISO 4649 [27] method A (non-rotating test piece configuration). The test specimens were cylindrical, with a diameter of 16 mm and a height of 6 mm. The percentage ratio of the volume loss of the test rubber to that of a standard rubber was calculated to obtain the abrasion resistance index (ARI, %). Three samples were tested for each compound, and the average volume loss from these samples was used to determine the ARI.

## 3. Results and Discussion

### 3.1. Various Oil and Resin Ratios

Compounds with different ratios between the oil and resin were evaluated to investigate how the oil and resin influence the properties of the material. As a reference, a compound with 37.5 phr TDAE oil was evaluated. The selected oil and resin were Hexamoll DINCH and Escorez 5300. Due to the cycloaliphatic nature of both substances, it was expected that they would be compatible with each other, enhancing the influence on the final properties of the rubber compound. Additional characteristics of the two substances are displayed in Table 1. Three different ratios between the Hexamoll DINCH and Escorez 5300 were evaluated, namely 20:80, 50:50, and 80:20. Table 3 shows the rubber formulations of these compounds.

In addition to the plasticizer blends, a compound with 37.5 phr of Hexamoll DINCH (H100) and a compound with 37.5 phr Escorez 5300 (E100) were mixed and evaluated. These results are shown and discussed in the evaluation of the DMA measurements.

#### 3.1.1. Rheometer Curves and Payne Effect

**Figure 1:**
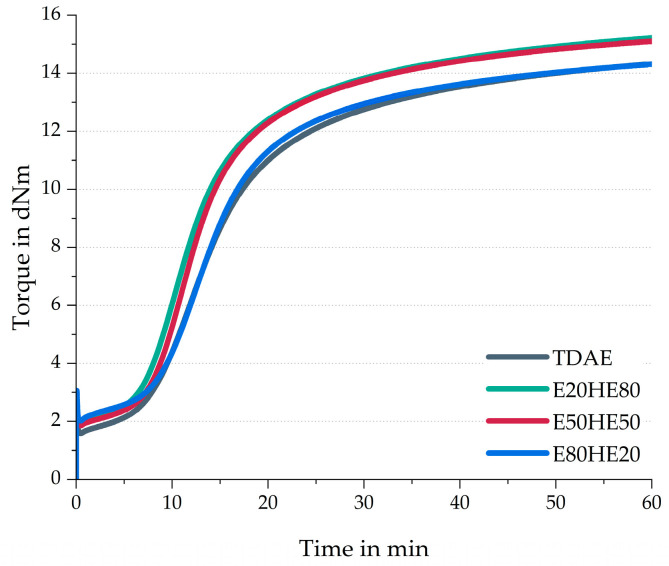
Rheometer curves of the compounds with various ratios between oil and resin.

Figure 1 depicts the rheometer curves of the compounds with various oil and resin ratios, and Table 4 contains additional curing characteristics like the scorch time (ts2), the optimum cure time (t90), maximum torque (MH), minimum torque (ML), and difference in torque (MH-ML).

Figure 1 and Table 4 reveal that the ML value is lowest for TDAE, while it is slightly higher for E20HE80 and E50HE50, and the highest value is obtained for E80HE20. The ML is related to the viscosity of the compound, where a higher ML indicates a higher compound viscosity [28]. Plasticizers are added to the compound to lower the viscosity of the compound and improve processing and dispersion [11]. Therefore, the high value obtained for the E80HE20 compound can be explained by the relatively high resin content, which has a higher molecular weight compared to the used oil (Table 1). In general, an increase in molecular weight is related to an increase in viscosity. Although the resin will change from a solid to a low viscous liquid at processing and curing temperatures [11], the viscosity is likely higher than the used di-ester oil at these temperatures.

A clear trend is visible between the oil and resin ratio and the scorch time. A higher concentration of oil, and a lower concentration of resin, results in a shorter scorch time. This can be explained by the concentration of ester-based oil in the compound. The ester groups might interact with the silica surface [29]. Due to these interactions, the silica surface is unable to absorb curing activators and accelerators, which become available for the vulcanization process. This could explain the faster scorch time, but also the higher maximum torque values for E20HE80 and E50HE50 because these are the two compounds with the highest concentration of ester-based oil. Usually, the maximum torque in the cure curve is correlated to polymer–polymer, polymer–filler, and filler–filler interactions [28]. As a result of more available curatives for the actual curing reaction in these compounds, more polymer–polymer interactions can be formed, causing an increase in the maximum torque.

**Figure 2:**
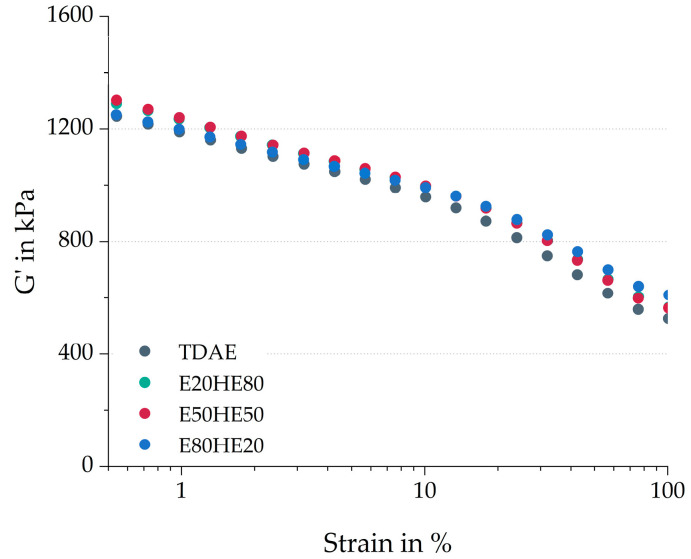
Cured Payne curves of the compounds with various ratios between oil and resin.

Figure 2 shows the storage modulus of the cured samples over a range of low to high strain values. The difference between the storage modulus at low and high strains is the Payne effect, in this study indicated with G’_0.56–100%_. The Payne effect is associated with the breakdown of filler–filler interactions and a lower Payne effect is usually related to an improved micro-dispersion of the filler in the compound [30]. The G’_0.56–100%_ and the storage modulus at 100% strain (G’_100%_) are displayed in Table 4.

Although the figure shows similar Payne effect curves for all four compounds, when considering the values in the table, slight differences are visible. Especially E80HE20 results in different values, with a lower cured Payne effect, but a higher storage modulus at 100% strain. The lower cured Payne effect can be caused by the higher molecular weight of the resin, leading to higher shear forces during mixing, improving the silica dispersion. A similar effect was observed by Bernal-Ortega et al. [16], who used the Escorez 5300 resin in combination with TDAE oil in a silica-filled S-SBR/BR compound. The slightly higher Payne effect values for E20HE80 and E50HE50 indicate slightly more filler–filler interactions, which might also contribute to the higher maximum torque values obtained for these two compounds in the cure curves (Figure 1) [28]. However, the differences are small, and therefore it is more likely that the higher maximum torque is caused by an increase in crosslink density. Equilibrium swelling is commonly used to evaluate the crosslink density of rubber samples. The widely used procedure, as described by Bernal-Ortega et al. [31], proved unsuitable for this study. It was observed that the resin did not dissolve during the initial extraction step using acetone, while the oil was effectively removed. As a result, the measurements were significantly affected by the presence of the undissolved resin, leading to invalid outcomes. Additional trials without the extraction step were performed, but these trials also resulted in non-reproducible and invalid values.

The storage modulus at 100% strain is influenced by the polymer network, the hydrodynamic effects, and in-rubber structures [30]. In this study, the higher G’_100%_ is most likely influenced by an increase in in-rubber structures, for example, due to more polymer–filler interactions. Bernal-Ortega et al. [17] studied the use of bio-based resins in silica-filled S-SBR/BR compounds and concluded that the pure resins could act as “glue” between the fillers, making the filler–filler interactions stronger. A similar phenomenon could occur when using the E80HE20 blend. This plasticizer blend might act as “glue” between the polymer and the filler, making these interactions stronger, and increasing the G’_100%_ slightly. This effect is less pronounced for the other plasticizer blends, due to the higher oil content.

Based on all characteristics obtained from the cure curves and the cured Payne measurements (Table 4), it can be concluded that the values of the E20HE80 and E50HE50 compounds are similar to each other, while the E80HE20 blend shows deviating behavior. This could indicate that the influence of the oil is more dominant when a similar or higher amount of oil is used compared to the resin. Because Hexamoll DINCH is more polar than Escorez 5300, as can be concluded from the δ_p_ values in Table 1, more interactions between the oil and the other rubber ingredients are expected. When the concentration of resin becomes higher, and the concentration of oil lower, the influence of the polar oil on the material properties becomes less substantial. Nonetheless, the three plasticizer blends lead to relatively similar curing behavior and cured Payne effect as the reference compound with TDAE.

#### 3.1.2. Stress–Strain Behavior and Tire Performance

**Figure 3:**
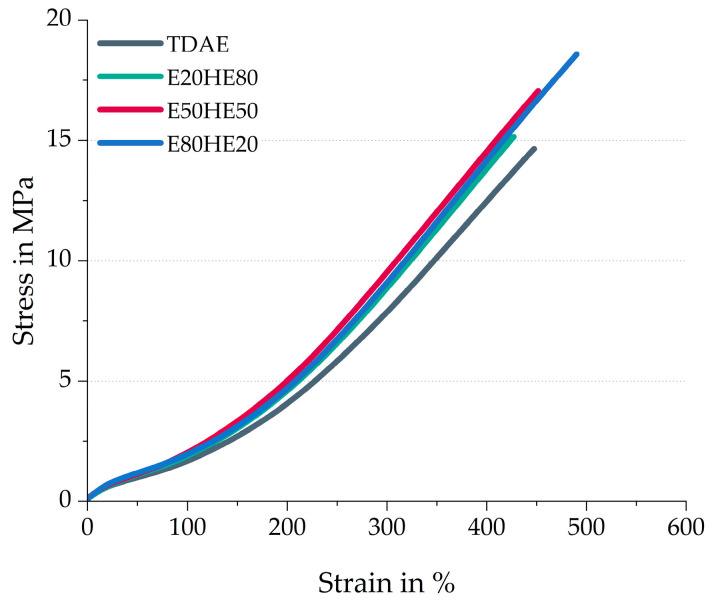
Stress–strain behavior of the compounds with various ratios between oil and resin.

Figure 3 shows the stress–strain behavior of the compounds. In Table 5 the stress–strain properties like the modulus at 100% strain (M100) and 300% strain (M300), the tensile strength (Ts), and the elongation at break (E_ab_) are presented. This table also contains the hardness values of the compounds.

An increasing value of tensile strength and elongation at break is obtained when increasing the resin content and decreasing the oil content in the compound, as shown in Figure 3. The stress–strain behavior was measured at room temperature. When mixing the pure Escorez 5300 in various ratios with the pure Hexamoll DINCH above the softening point of the resin, and cooling it down to room temperature, the mixture stays in a liquid form and no phase separation was observed. However, the ratio between oil and resin did influence the viscosity of the mixture, where a higher resin content resulted in a more viscous liquid than the mixture with a higher oil content. The higher viscosity of the E80HE20 plasticizer blend likely results in higher shear forces during mixing, enhancing the filler dispersion. Because of this, more filler–polymer interactions might be formed, increasing the strength of the compound.

All three plasticizer blends result in higher M100 and M300 values, compared to the reference compound with TDAE oil. This is presumably caused by the increased polymer–polymer and polymer–filler interactions in these three plasticizer blend compounds, which is in line with the higher G’_100%_ observed in the cured Payne effect measurements.

The hardness of the compounds as displayed in Table 5 is similar for all compounds, because the values lie within the measurement error of the device (±2 Shore A). Therefore, it is not possible to conclude that there is a significant hardness increase when using more resin in the plasticizer blend.

Based on the stress–strain behavior and hardness, the E50HE50 and E80HE20 show more similar values to each other, compared to the E20HE80 blend. This is in contrast with the cure characteristics and cured Payne effect, where the E50HE50 blend showed more similarities with E20HE80. A possible explanation is that at temperatures significantly higher than the glass transition temperature of the plasticizers, both plasticizers are liquid and behave similarly. This is evident in measurements of the cure characteristics at 160 °C and the Payne effect at 100 °C. At these high temperatures, the chemical differences between the plasticizers, such as the polarity of the di-ester oil, play a larger role than the physical state. At room temperature, the oil is still above the T_g_, while the resin is below the T_g_ (Table 1). Therefore, the physical state of the resin differs and has a dominant influence on the final properties of the rubber compound.

Table 6 shows the normalized ARI values of the three plasticizer blends, compared to the reference with TDAE. All three plasticizer blends result in a lower resistance against wear, which has a negative influence on the durability of the tread compound. No clear trend between the oil and resin ratio and the wear resistance can be observed.

**Figure 4:**
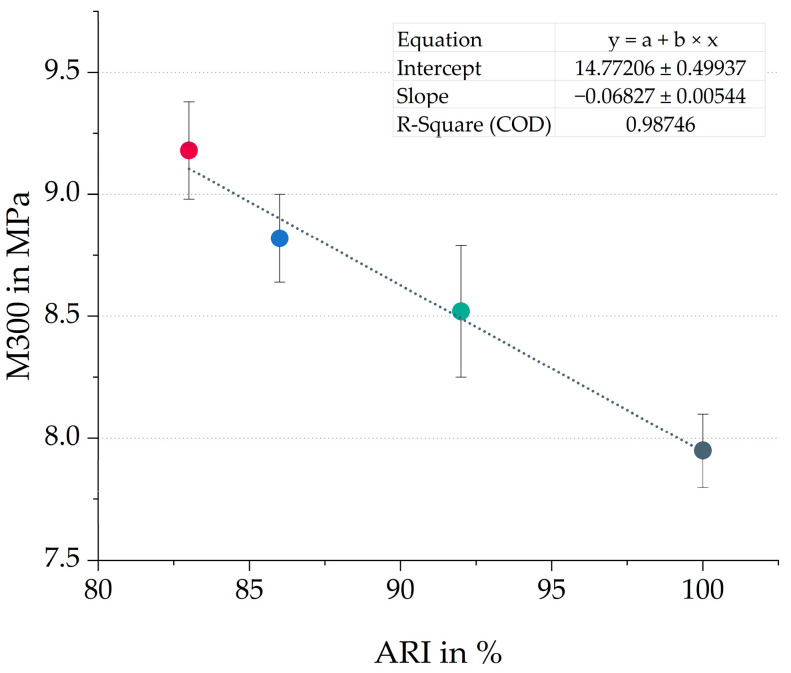
Correlation between wear resistance (ARI) and modulus at 300% strain (M300) of all four compounds. The colors correspond to the evaluated compounds (gray = TDAE, green = E20HE80, red = E50HE50 and blue = E80HE20).

However, an inverse linear correlation between the ARI and the M300 is obtained for the compounds evaluated in this study, as shown in Figure 4. Although typically an increase in stiffness leads to improved wear resistance, excessive stiffness can lead to the opposite effect [32]. The material might be slightly more brittle, which makes it more prone to the formation of cracks.

**Figure 5:**
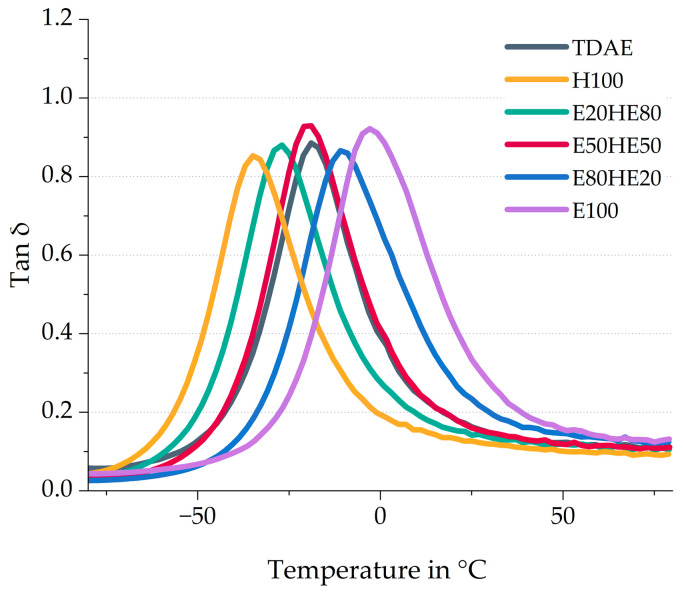
Tan δ curves of the compounds with various ratios between oil and resin.

Figure 5 depicts the tan δ curves of the compounds with various plasticizer blends. The peak position can be related to the glass transition temperature of the compound [33]. To understand the influence of the T_g_ of the pure oil and resin on the compound, the compounds with 37.5 phr of Hexamoll DINCH (H100), and the compound with 37.5 phr of Escorez 5300 (E100) are also included in Figure 5. Moreover, the tire tread indicators like the wet grip (tan δ at 0 °C) and rolling resistance (tan δ at 60 °C), as well as the glass transition temperature (T_g_), are displayed in Table 7.

**Figure 6:**
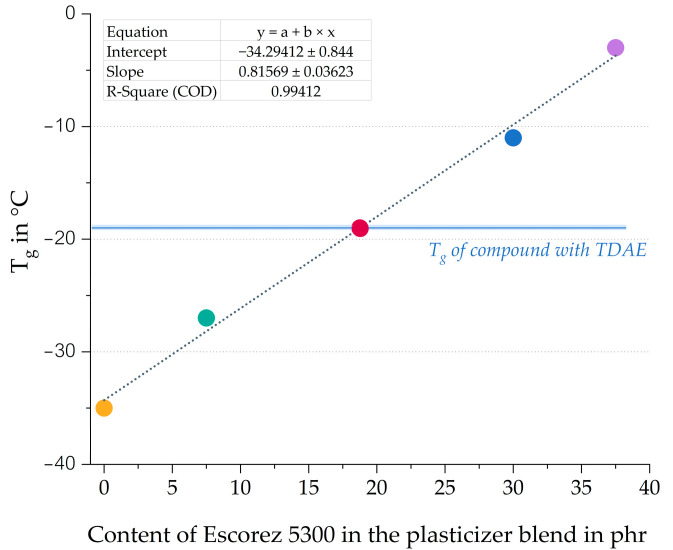
Glass transition temperature obtained with DMA plotted against the Escorez 5300 content. All compounds contain 37.5 phr of plasticizer (Escorez 5300 and Hexamoll DINCH) as shown in Table 3. The colors of the data points correspond to the evaluated compounds and match those used in Figure 5.

A higher content of oil results in a lower T_g_ value, while a higher resin concentration leads to a higher T_g_. These shifts in T_g_ are even more pronounced for the H100 and E100 compounds. Table 1 displays the T_g_ of the pure plasticizers, and it can be observed that the T_g_ of the di-ester oil is much lower than that of the resin. Because a relatively large part of the compound consists of a plasticizer, the T_g_ of the pure plasticizer strongly influences the T_g_ of the final compound. This explains why the T_g_ of the compound with a high oil content is much lower than the T_g_ of the compounds with high resin content. Figure 6 shows the correlation graph of the T_g_ obtained with DMA measurements against the Escorez 5300 content.

Based on Figure 6, it becomes clear that there is a linear correlation between the T_g_ of the compounds and the plasticizer blend ratios. Using various oil and resin blends makes it possible to modify the T_g_ of the vulcanizate, which is an efficient approach for tailoring the glass transition temperature according to specific customer requirements and the desired properties of the final product. Furthermore, the 50/50 blend of Hexamoll DINCH and Escorez 5300 results in a similar tan δ peak position as the reference compound with TDAE.

The wet grip and rolling resistance indicators can be obtained from the tan δ curves: the tan δ at low temperatures (0 to 20 °C) indicates the wet grip, and the tan δ at higher temperatures (40 to 80 °C) indicates the rolling resistance [34]. In this study, the tan δ at 0 °C and 60 °C are used as wet grip and rolling resistance indicators, respectively, and displayed in Table 7. A high tan δ at 0 °C and low tan δ at 60 °C are preferred. It needs to be considered that the wet grip indicator is less reliable than the rolling resistance indicator. The wet grip evaluated with Dynamic Mechanical Analysis (DMA) measurements does not always correlate with the wet grip obtained during actual breaking tests, especially when resins are introduced [35]. Due to the influence of the resin on the T_g_, shifting it to temperatures closer to 0 °C, the wet grip rises as well. Because the rolling resistance indicator is measured at temperatures further away from the T_g_, this indicator is observed to be less influenced by the shift in T_g_ and is therefore more reliable. Additional measurements are needed to get better insight into the tire properties of the compounds. However, in this study, the DMA indicators are shown to give a first indication. Of the three plasticizer blends, the E80HE20 results in the highest wet grip indicator, due to the high resin content. However, this compound also results in the highest rolling resistance indicator. The best balance in tire performance properties obtained from the tan δ curves is obtained for the E50HE50 compound.

**Figure 7:**
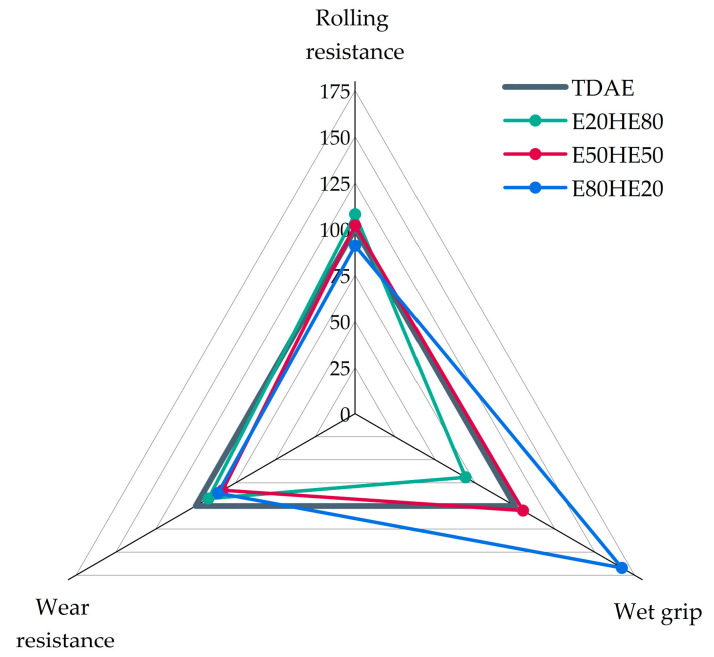
Magic triangle of the compounds with various oil and resin ratios.

To summarize the most important tire properties, the magic triangle of tire performance is displayed in Figure 7. In this figure, the values are normalized, and the results obtained for the reference compound with TDAE are set to 100. For all three properties, a higher value is preferred.

Based on the results in Figure 7 it can be concluded that the various oil and resin blend ratios influence the wet grip to the greatest extent. Using a blend with a higher resin content, in this case 80%, results in a severe increase in the wet grip indicator. However, decreased values for the rolling resistance and wear resistance are obtained for this blend. The best balance can be observed for the 50/50 blend, which results in a slightly higher wet grip, a similar rolling resistance, and only slightly decreased wear resistance, compared to the reference compound. The blend ratio with only 20% of resin shows relatively good results for the wear resistance and rolling resistance, but a much lower value for the wet grip indicator. Based on these results and the T_g_ values, it can be concluded that the replacement of TDAE with 18.75 phr of Hexamoll DINCH and 18.75 phr of Escorez 5300 is the most feasible.

### 3.2. Plasticizer Blends with Resin and Different Di-Ester Oils

To further evaluate the plasticizer blends, three different di-ester oils were selected to blend with Escorez 5300. All oils are di-ester oils, with different chemical structures between the two ester groups. These structures and additional characteristics are displayed in Table 1. The oils were blended with the resin in a 50:50 ratio, because this was the most successful plasticizer blend ratio based on the tire properties, and this ratio resulted in a similar glass transition temperature as the reference compound with TDAE. In addition to the more conventional concentration of 37.5 phr plasticizer, the compounds were mixed with high plasticizer concentrations of 80 phr (40 phr of oil, and 40 phr of resin). This way, the influence of the used plasticizer blends on the compound properties was assumed to become more visible. The rubber formulations of both the conventional concentration plasticizer and the high concentration are presented in Table 8. In the figures and tables presented in this chapter, the high-plasticizer compounds (80 phr) are indicated with an (H).

For the high-concentration plasticizer compounds, the resin was added in three separate portions to the compound. The portions were added in the first stage after 1 min, 2.5 min, and 4 min of mixing. Furthermore, the mixing procedure was kept the same as shown in Table 2.

#### 3.2.1. Rheometer Curves and Payne Effect

**Figure 8:**
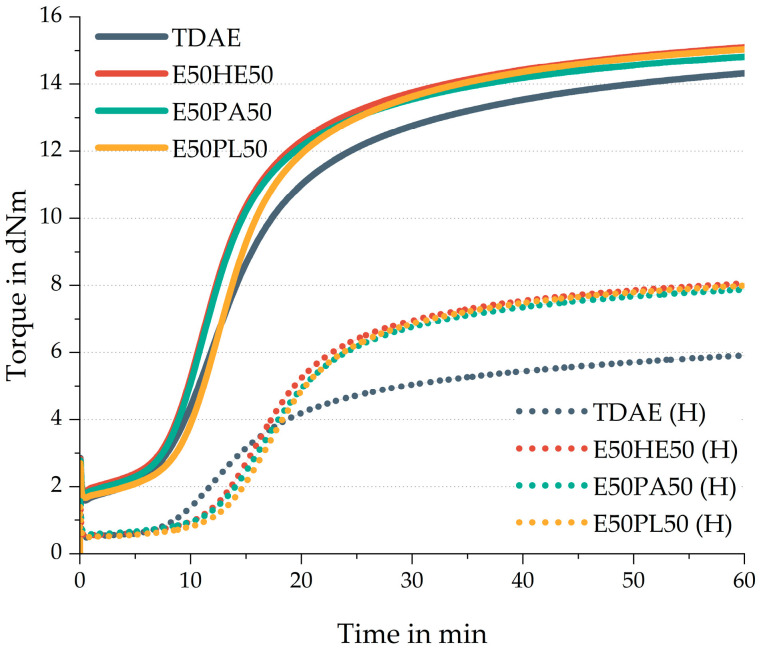
Rheometer curves of the compounds with resin and different di-ester oils. Compounds with high concentrations of plasticizer are indicated with (H).

In Figure 8 the rheometer curves of the compounds are shown. The solid lines indicate the conventional plasticizer concentrations, while the high-concentration plasticizer compounds are indicated with the dotted lines. Table 9 displays the additional curing characteristics.

All plasticizer blends show similar cure curves and curing characteristics compared to each other. When focusing on the compounds with 37.5 phr, the scorch time of TDAE is similar to the scorch times observed for the compounds with plasticizer blends. When comparing this characteristic in the high-concentration plasticizer compounds, a shorter scorch time is obtained for TDAE (H) than for the blends. According to the characteristics of TDAE as described by Rathi [12], this oil contains a small content of 0.8 wt% of sulfur. At lower concentrations, this sulfur in TDAE does not have a significant effect on the curing reaction. However, when higher concentrations of TDAE oil are used, relatively more sulfur is added to the compound, increasing the scorch time slightly.

Compared to the reference compound with TDAE, the plasticizer blends all show higher maximum torque values. This could be related to a higher crosslink density for these compounds. When evaluating the 80 phr plasticizer compounds, the difference between the maximum torque of the 50/50 blends and the reference is even more severe. This could be due to two influences. On the one hand, it might be that the plasticizer blends enhance the curing reaction. The ester groups in the plasticizer blends could interact with the silica surface, shielding this filler, which might result in more curing ingredients available in the compound. However, it would be expected that this would also result in a shorter scorch time, which is not observed for the high-concentration plasticizer blend compounds. On the other hand, TDAE could interfere with the curing process, for example by reacting with the sulfur and therefore hindering the vulcanization process. A reason for this can be that TDAE might contain some unsaturation, which can interact with the sulfur, leading to a lower crosslink density [13].

In general, much lower torque values are obtained for the high plasticizer compounds than for the compound with 37.5 phr plasticizer. When increasing the plasticizer concentration without changing the quantities of the other ingredients there are relatively fewer polymer, filler, and curing chemicals available in the compound. This influences the rubber network structure of the material, for example, fewer crosslinks can be formed.

**Figure 9:**
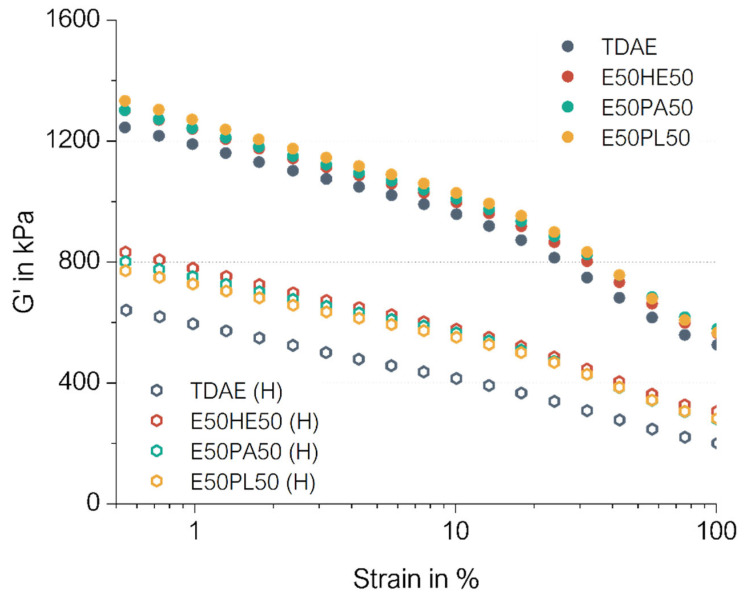
Cured Payne curves of the compounds with resin and different di-ester oils. Compounds with high concentrations of plasticizer are indicated with (H).

The cured Payne effect curves are depicted in Figure 9. In this figure, the darker-colored dots are related to the compounds with conventional concentrations of plasticizer. The hollow hexagon-shaped markings indicate the results of the high-plasticizer compounds. The cured Payne effect values and storage moduli at 100% strain can be seen in Table 9.

The cured Payne effect of the compounds with 37.5 phr of plasticizers are all similar. A slight shift to higher G’ values compared to TDAE can be observed which is likely due to a slightly higher crosslink density. These results are in agreement with the maximum torque values obtained from the rheometer curves (Figure 8).

Comparing the cured Payne effect of the 37.5 phr plasticizer compounds with the 80 phr plasticizer compound, it becomes clear that much lower values are obtained for the high-concentration plasticizer compounds. This can be explained by the fact that the compounds contain relatively much more plasticizer, and therefore less filler and polymer. This results in less filler–filler, filler–polymer, and polymer–polymer interactions, resulting in lower storage modulus values, as well as lower cured Payne effect.

The compounds with a high content of plasticizer blends show a clear shift in the G’ throughout the measurement, compared to TDAE (H). As explained previously, TDAE likely interferes with the curing reaction by interacting with the curing ingredients. Therefore, a lower crosslink density is obtained, leading to lower G’ values during the applied strain sweep. This effect is more pronounced when high concentrations of the TDAE are used.

#### 3.2.2. Stress–Strain Behavior and Tire Performance

**Figure 10:**
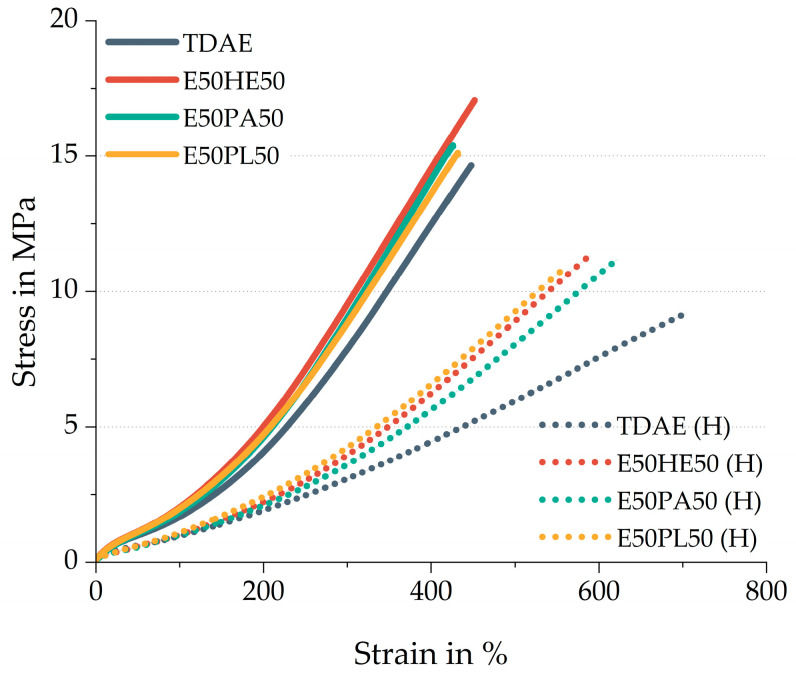
Stress–strain behavior of the compounds with resin and different di-ester oils. Compounds with high concentrations of plasticizer are indicated with (H).

The stress–strain behavior of the compounds is shown in Figure 10. The solid lines indicate the compounds with 37.5 phr of plasticizer, while the dotted lines indicate the high-plasticizer compounds. Table 10 contains additional stress–strain properties and the hardness of the compounds.

The 37.5 phr plasticizer compounds with 50/50 oil and resin blends show all three slightly higher modulus values compared to the reference compound with TDAE oil. These results are caused by the possible higher crosslink density and higher polymer–filler interactions, which is consistent with the observed findings from the curing behavior and cured Payne effect.

The E50HE50 shows the most distinct behavior compared to the two other blends, with a higher tensile strength and elongation at break. Because the results of the rheometer curves and the cured Payne effect are similar, it is not likely that the E50HE50 results in significant differences in network structure. The higher tensile strength and elongation at break might be due to an improved compatibility between the oil and the resin. Comparing the Hansen Solubility Parameter values in Table 1 of the resin with the three di-ester oils, it can be concluded that the Hexamoll DINCH results in a more similar δd and δh in comparison to Escorez 5300.

Comparing the M100 and M300 values of the high-concentration plasticizer compounds, the difference between the plasticizer blends and TDAE becomes more pronounced. This further illustrates that the plasticizer blends enhance the reinforcement of the vulcanizates. As previously mentioned, this can be explained by the possible higher crosslink density and the higher polymer–filler interactions.

The low tensile strength and high elongation at break obtained for TDAE (H) can be explained by the lower crosslink density of this sample, as explained in the previous subchapter. These stress–strain results are in line with the rheometer curves and cured Payne effect obtained for this compound.

**Figure 11:**
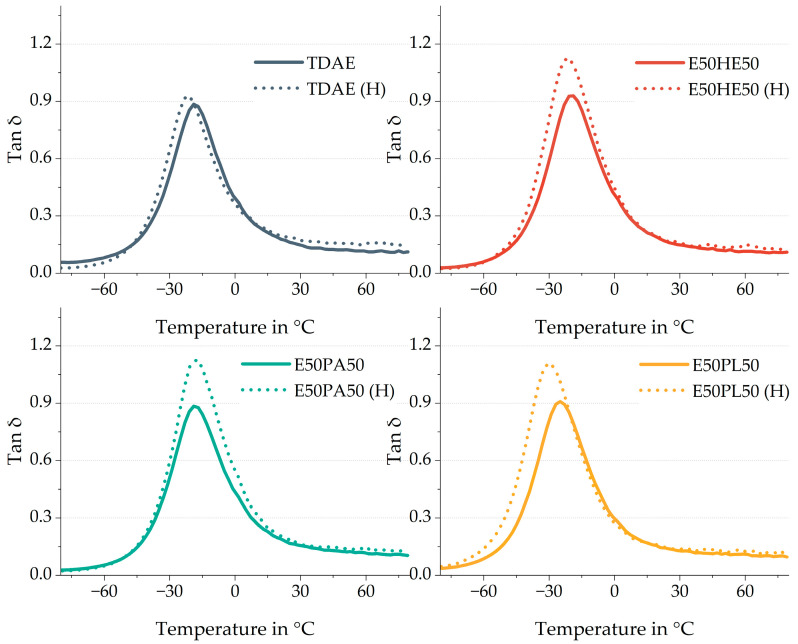
Tan δ curves of the compounds with resin and different di-ester oils. Compounds with high concentrations of plasticizer are indicated with (H).

Figure 11 shows the tan δ curves of the analyzed 50/50 blends, compared to the reference with TDAE. The dotted lines show the high-concentration plasticizer compounds. The values of the tire performance properties as well as the T_g_ are shown in Table 11.

One of the main differences that can be observed in Figure 11 is the height of the tan δ. For the high-concentration plasticizer blends, the peak is higher with values of approximately 1.1, compared to the compounds with the conventional amount of plasticizer which show tan δ values of approx. 0.9. The tan δ is the ratio between the loss modulus E″ and the storage modulus E′. A higher tan δ often indicates higher dissipation behavior, while a lower tan δ is related to a higher elastic behavior [33]. Increasing the plasticizer results in a larger viscous and dissipating part, increasing the loss modulus.

The T_g_ is also influenced by the higher concentration of plasticizer in the compounds with the E50PA50 and E50PL50 blends. This effect is most pronounced for E50PL50, which can be explained by the lower T_g_ of the pure Plastomoll DOA di-ester oil (Table 1). Because this oil inhibits a lower T_g_, and the amount of oil is increased, the influence of the oil on the compound T_g_ becomes larger. For the E50PA50 a similar effect is obtained. However, the higher T_g_ of the pure Palationol 10P results in a slightly higher T_g_ of the compound compared to the TDAE (H) compound. E50HE50 shows the same T_g_ as TDAE when using 37.5 phr of plasticizer and when using 80 phr. This indicates that the combination of 50% Hexamoll DINCH and 50% Escorez 5300 results in the most similar T_g_ as TDAE.

Table 12 includes the normalized ARI values of the compounds with 37.5 phr of plasticizer. Based on these results, it becomes clear that the best wear resistance is obtained for the compound with the blend between Escorez 5300 and Plastomoll DOA. Veith [36] observed a linear correlation between the glass transition temperature and the wear resistance of SBR/BR rubber blends. A lower T_g_ of the compound resulted in better wear resistance. Therefore, it is assumed that the improved wear resistance of the E50PL50 compound compared to the other two plasticizer blends is due to the lower T_g_ of this rubber material. The plasticizer blends with a more similar T_g_ to the reference compound, namely the E50HE50 and E50PA50, show worse wear resistance. Full replacement of the TDAE oil with Escorez 5300 resin in the study of Bernal-Ortega et al. [16] showed an improved abrasion resistance index. This is explained by the possible better filler dispersion due to the higher shear forces obtained when mixing with resin. In this study, the cured Payne effect of all 50/50 plasticizer blends is slightly higher, which might indicate a slight deterioration in the filler dispersion compared to TDAE. The worse filler dispersion could be a reason for the lower wear resistance. Moreover, the 50/50 plasticizer blends show higher moduli in the stress–strain curves and higher hardness values compared to TDAE. This slight increase in stiffness and hardness could be another cause for the decrease in wear resistance.

**Figure 12:**
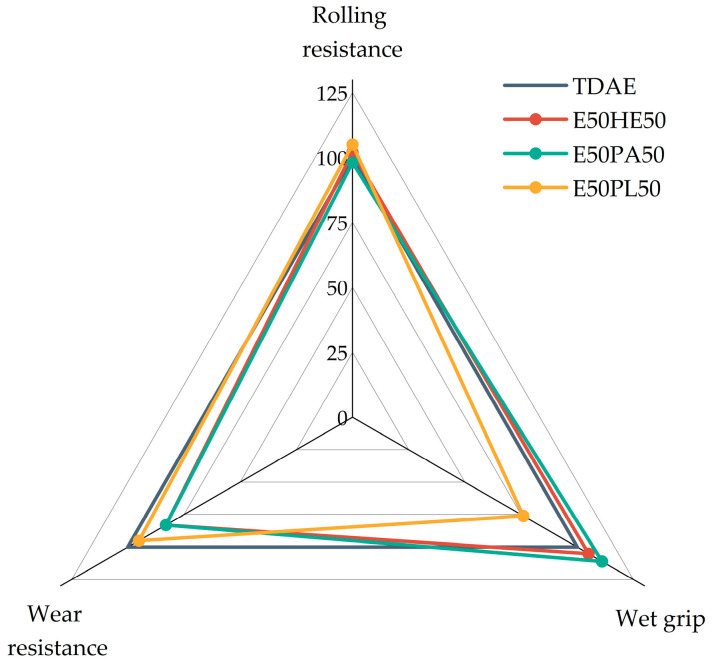
Magic triangle of compounds with resin and different di-ester oils.

The magic triangle of tire performance is displayed in Figure 12. The rolling resistance, wet grip, and wear resistance are normalized based on the reference compound with TDAE. In the figure, a higher value indicates an improved performance. Only the compounds with 37.5 phr of plasticizer are displayed in this figure because the high-concentration plasticizer compounds are not feasible for tire tread applications due to their poor mechanical properties.

Based on the magic triangle, it can be concluded that the E50PL50 compound shows the best rolling resistance and wear resistance, but the worst wet grip. The other two blends (E50HE50 and E50PA50) result in similar rolling resistance values as TDAE, slightly increased wet grip indicators, but lower wear resistance values. However, the differences are relatively small, and in general, the 50/50 blends result in similar values as the reference with TDAE, which is promising.

The differences between the chemical structures in the selected di-ester oils do not result in a severe influence on the final properties of the rubber compounds. It is more likely that the physical characteristics of the oils, like molecular weight and glass transition temperature, influence the slight differences obtained in this study. High-concentration plasticizer compounds show that the plasticizer blends possibly enhance the crosslink formation of the compounds, and result in improved reinforcement, compared to the compound with TDAE.

## 4. Conclusions

When using bio-based oils in rubber compounds, e.g., vegetable oils, the glass transition temperature is observed to shift towards lower temperatures. The opposite occurs when using resins. These shifts in T_g_ can influence the performance of the final rubber vulcanizate, which is not always advantageous. To find a plasticizer that is more sustainable, and results in a similar T_g_ to a compound with commonly used petroleum-based plasticizers, it was proposed to use an oil and resin plasticizer blend.

In the first part of this study, three different oil and resin blend ratios were used in a silica-filled S-SBR/BR compound, and compared to a reference compound with TDAE. The di-ester oil Hexamoll DINCH and resin Escorez 5300 were selected due to their cycloaliphatic nature. Various properties were evaluated, among which the curing characteristics, cured Payne effect, stress–strain behavior, hardness, viscoelastic behavior, and wear resistance. Most measurements showed relatively similar results for the compounds with plasticizer blends based on Hexamoll DINCH and Escorez 5300 compared to the compound with TDAE. However, a clear influence of the oil and resin blend ratio on the viscoelastic behavior of the material was observed. A correlation plot between the T_g_ of the compound and the Escorez 5300 concentration revealed a linear correlation. This suggests that the T_g_ of the compound can be easily modified using various plasticizer blend ratios, without influencing the curing behavior or strength of the material. This approach can be used to tailor the T_g_ of the rubber compound based on the application of the material. However, the various plasticizer blends do influence the laboratory predictors for the wet grip and rolling resistance. The best balance in properties was obtained for the 50/50 blend.

In the second part of this work, various di-ester oils were selected and used in 50/50 plasticizer blends with Escorez 5300. Compounds with more conventional concentrations of plasticizer (37.5 phr) as well as compounds with high plasticizer content (80 phr) were mixed. All three plasticizer blends showed similar results, except for some slight differences in the E50PL50 compounds. This could be explained by the lower molecular weight and lower T_g_ of the pure di-ester oil. The differences between the plasticizer blends and TDAE in the high-concentration plasticizer compounds were more severe. These compounds showed even more clearly that all three blends enhance the crosslinking formation and result in an improved reinforcement compared to the compound with TDAE. Among the three blends, the E50HE50 blend showed the most similar T_g_ in both plasticizer concentrations.

Because Hexamoll DINCH can be created via the BioMass Balance approach, the E50HE50 blend is 50% bio-based. This study demonstrates that using the E50HE50 blend as a replacement for TDAE results in similar performance while increasing the sustainability of the compound.

This study primarily focused on evaluating the performance characteristics of the final rubber compound. However, it is recognized that replacement of the plasticizer might influence the processability of the compound. Therefore, it is recommended that future research contains a more detailed analysis of processing properties, for example, the Mooney viscosity.

## Figures and Tables

**Table 1:** Main characteristics according to the technical data sheets of the studied plasticizers.

Ingredient	Mw in g/mol	ρ in g/cm^3^	T_g_ * in °C	HSP ** in MPa^1/2^	Chemical Structure
TDAE	x	0.95	−52	δ_D_ = 15.2 δ_P_ = 11.2 δ_H_ = 1.0 δ_tot_ = 18.9	Carbon distribution [12]: C_aromatic_ = 25 wt% C_napthene_ = 30 wt% C_paraffin_ = 45 wt%
Hexamoll^®^ DINCH	425	0.95	−90	δ_D_ = 16.1 δ_P_ = 1.9 δ_H_ = 2.6 δ_tot_ = 16.4	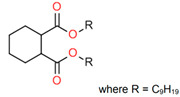
Palatinol^®^ 10P	447	0.96	−79	δ_D_ = 16.8 δ_P_ = 4.2 δ_H_ = 2.3 δ_tot_ = 17.4	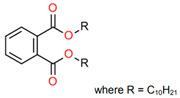
Plastomoll^®^ DOA	371	0.92	−106	δ_D_ = 16.2 δ_P_ = 2.7 δ_H_ = 3.4 δ_tot_ = 16.7	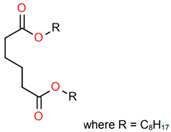
Escorez™ 5300	670	1.01	45	δ_D_ = 17.9 δ_P_ = 0.1 δ_H_ = 0.1 δ_tot_ = 17.9	Cycloaliphatic hydrocarbon resin

* Obtained with Differential Scanning Calorimetry measurements using the DSC 214 Polyma with a heat rate of 10 °C/min. ** Calculated with the Hansen Solubility Parameter in Practice (HSPiP) software (5th edition, version 5.4.04), except for the values of TDAE, which were obtained from [24]. In addition, the HSP values for the S-SBR/BR blend were calculated (δ_D_ = 18.5 MPa^1/2^, δ_P_ = 0.9 MPa^1/2^, δ_H_ = 1.7 MPa^1/2^, δ_tot_ = 18.9 MPa^1/2^).

**Table 2:** Mixing procedure.

Time	Action
**m:s**	**Stage 1:** *pre-heating 80 °C, 70 rpm, fill factor 72%*
0:00	Addition of rubber
1:00	Addition of 2/3 silica, 2/3 silane
2:30	Addition of 1/3 silica, 1/3 silane, ZnO, Stearic acid, and plasticizer
4:00	15-s ramp sweep
4:15	Increase of torque (increase temperature to 130 °C)
7:00	Stop mixing (reaching 140 °C)
**m:s**	**Stage 2:** *pre-heating 80 °C, 80 rpm, fill factor 69%*
0:00	Addition of elastomer masterbatch
0:50	Addition of DPG
1:00	Increase of torque (increase temperature to 130 °C)
5:00	Stop mixing (reaching 140 °C)
**m:s**	**Stage 3:** *pre-heating 50 °C, 50 rpm, fill factor 66%*
0:00	Addition of elastomer masterbatch, curatives (sulfur, and TBBS)
3:00	Stop mixing

**Table 3:** Rubber formulations for compounds with various ratios between oil and resin.

Compounds	S-SBR in phr	BR in phr	Silica in phr	TDAE in phr	Hexamoll DINCH in phr	Escorez 5300 in phr
TDAE	80	20	80	37.5	X	X
E20HE80	80	20	80	X	30	7.5
E50HE50	80	20	80	X	18.75	18.75
E80HE20	80	20	80	X	7.5	30

Furthermore, the compounds consisted of the same quantities of the remaining ingredients: 6.2 phr TESPD, 2.5 phr stearic acid, 2.5 phr zinc oxide, 1.4 phr sulfur, 2.0 phr TBBS, and 1.5 phr DPG.

**Table 4:** Curing characteristics and Payne effect data of the compounds with various ratios between oil and resin.

Compounds	ts2 in min	t90 in min	MH in dNm	ML in dNm	MH-ML in dNm	G’_0.56–100%_ in kPa	G’_100%_ in kPa
TDAE	8.9	33.1	14.3	1.6	12.7	719	525
E20HE80	7.9	30.6	15.2	1.9	13.3	724	566
E50HE50	8.6	30.3	15.1	1.9	13.2	738	564
E80HE20	9.5	31.5	14.3	2.0	12.3	640	610

**Table 5:** Stress–strain data and hardness values of the compounds with various ratios between oil and resin.

Compounds	Hardness in Shore A	M100 in MPa	M300 in MPa	E_ab_ in %	Ts in MPa
TDAE	52 ± 0	1.71 ± 0.04	7.95 ± 0.15	460 ± 30	15.2 ± 1.4
E20HE80	51 ± 0	1.82 ± 0.09	8.52 ± 0.27	440 ± 20	15.0 ± 1.0
E50HE50	54 ± 0	1.94 ± 0.05	9.18 ± 0.20	450 ± 10	16.3 ± 0.6
E80HE20	54 ± 0	1.94 ± 0.05	8.82 ± 0.18	490 ± 30	17.9 ± 1.5

**Table 6:** Normalized abrasion resistance index values of the compounds with various ratios between oil and resin.

Compounds	ARI in %
TDAE	100
E20HE80	92
E50HE50	83
E80HE20	86

**Table 7:** Tire properties of the compounds with various ratios between oil and resin.

Compounds	T_g_ in °C	tan δ at 60 °C	tan δ at 0 °C
TDAE	−19	0.117	0.406
E20HE80	−27	0.114	0.287
E50HE50	−19	0.115	0.427
E80HE20	−11	0.136	0.692

**Table 8:** Rubber formulations of the compounds with resin and different di-ester oils.

Compounds	S-SBR in phr	BR in phr	Silica in phr	TDAE in phr	Hexamoll DINCH in phr	Palatinol 10P in phr	Plastomoll DOA in phr	Escorez 5300 in phr
TDAE	80	20	80	37.5	X	X	X	X
E50HE50	80	20	80	X	18.75	X	X	18.75
E50PA50	80	20	80	X	X	18.75	X	18.75
E50PL50	80	20	80	X	X	X	18.75	18.75
TDAE (H)	80	20	80	80	X	X	X	X
E50HE50 (H)	80	20	80	X	40	X	X	40
E50PA50 (H)	80	20	80	X	X	40	X	40
E50PL50 (H)	80	20	80	X	X	X	40	40

Furthermore, the compounds consisted of the same quantities of the remaining ingredients: 6.2 phr TESPD, 2.5 phr stearic acid, 2.5 phr zinc oxide, 1.4 phr sulfur, 2.0 phr TBBS, and 1.5 phr DPG.

**Table 9:** Curing characteristics and Payne effect data of the compounds with resins and different di-ester oils. Compounds with high concentrations of plasticizer are indicated with (H).

Compounds	ts2 in min	t90 in min	MH in dNm	ML in dNm	MH-ML in dNm	G’_0.56–100%_ in kPa	G’_100%_ in kPa
TDAE	8.9	33.1	14.3	1.6	12.7	719	525
E50HE50	8.6	30.3	15.1	1.9	13.2	738	564
E50PA50	8.6	29.6	14.8	1.8	13.0	723	579
E50PL50	9.7	30.8	15.0	1.7	13.4	766	567
TDAE (H)	13.0	37.6	5.9	0.5	5.4	440	201
E50HE50 (H)	14.7	35.1	8.1	0.5	7.5	526	306
E50PA50 (H)	15.2	35.6	7.9	0.6	7.3	522	282
E50PL50 (H)	15.7	35.2	8.0	0.5	7.5	489	280

**Table 10:** Stress–strain data and hardness values of the compounds with resin and different di-ester oils Compounds with high concentrations of plasticizer are indicated with (H).

Compounds	Hardness in Shore A	M100 in MPa	M300 in MPa	E_ab_ in %	Ts in MPa
TDAE	52 ± 0	1.71 ± 0.04	7.95 ± 0.15	460 ± 30	15.2 ± 1.4
E50HE50	54 ± 0	1.94 ± 0.05	9.18 ± 0.20	450 ± 10	16.3 ± 0.6
E50PA50	54 ± 0	1.94 ± 0.07	9.20 ± 0.09	430 ± 20	15.4 ± 0.9
E50PL50	54 ± 0	1.93 ± 0.05	8.85 ± 0.22	430 ± 10	14.9 ± 0.5
TDAE (H)	33 ± 0	0.93 ± 0.05	3.00 ± 0.12	700 ± 20	9.0 ± 0.3
E50HE50 (H)	39 ± 1	1.04 ± 0.05	4.05 ± 0.10	580 ± 20	11.1 ± 0.4
E50PA50 (H)	40 ± 0	1.03 ± 0.05	3.75 ± 0.19	620 ± 30	10.9 ± 0.7
E50PL50 (H)	39 ± 0	1.05 ± 0.04	4.15 ± 0.09	550 ± 30	10.6 ± 0.7

**Table 11:** Tire properties of the compounds with resin and different di-ester oils. Compounds with high concentrations of plasticizer are indicated with (H).

Compounds	T_g_ in °C	tan δ at 60 °C	tan δ at 0 °C
TDAE	−19	0.117	0.406
E50HE50	−19	0.115	0.427
E50PA50	−19	0.120	0.453
E50PL50	−25	0.111	0.309
TDAE (H)	−21	0.151	0.377
E50HE50 (H)	−21	0.138	0.463
E50PA50 (H)	−19	0.139	0.571
E50PL50 (H)	−31	0.130	0.285

**Table 12:** Normalized abrasion resistance index of the compounds with resin and different di-ester oils.

Compounds	ARI in %
TDAE	100
E50HE50	83
E50PA50	83
E50PL50	95

## Data Availability

The raw data supporting the conclusions of this article will be made available by the authors on request.
